# Supplementary figures and images for: Estrogen regulates sex-specific localization of regulatory T cells in adipose tissue of obese female mice
Source: PLoS One. 2020 Apr 2;15(4):e0230885. doi: 10.1371/journal.pone.0230885 (PMC7117686; doi:10.1371/journal.pone.0230885)

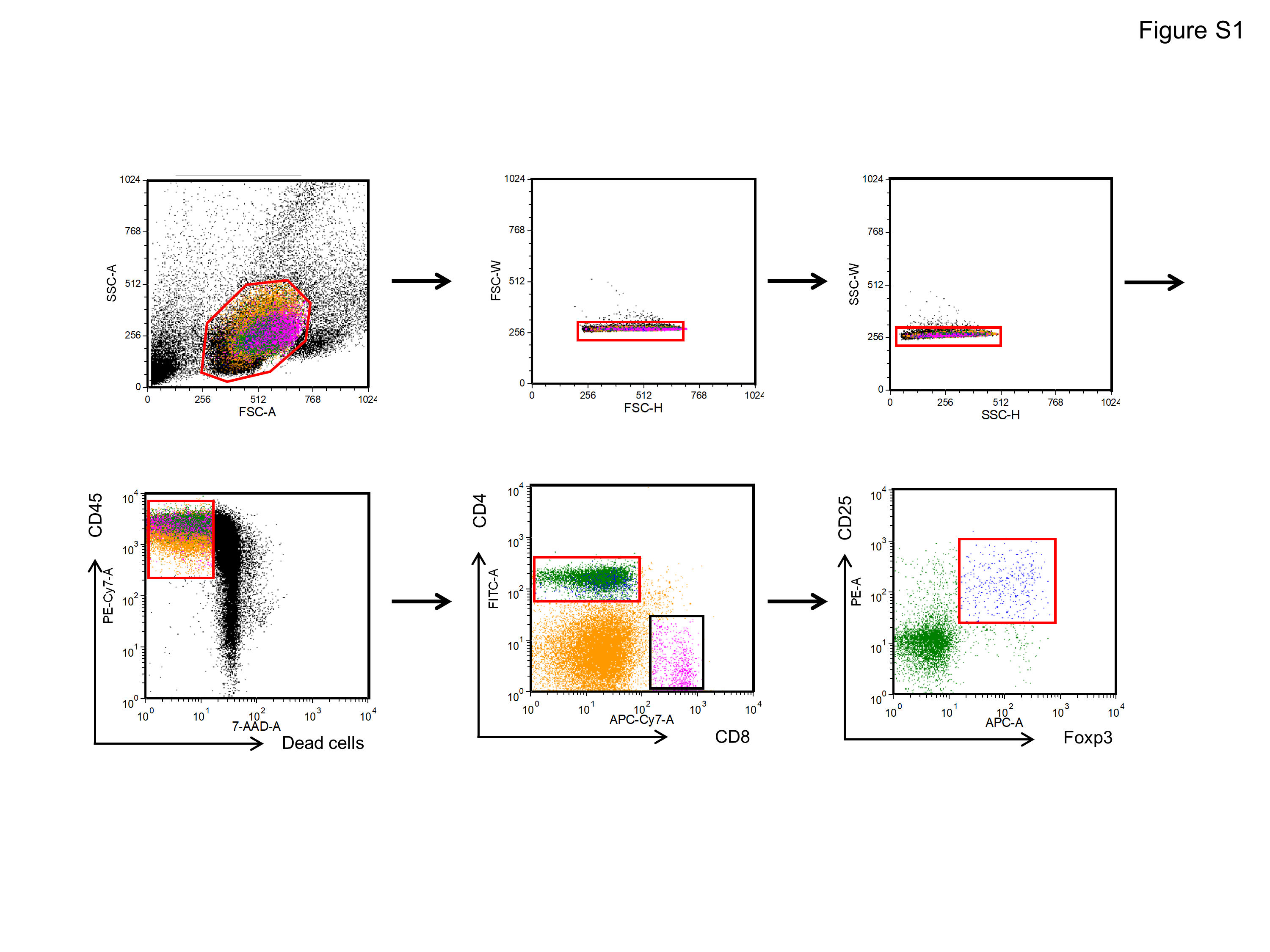

Supplement: S1 Fig — Representative plots of flow cytometry showing the gating strategy for identifying Tregs. (TIF) [file pone.0230885.s001.tif]

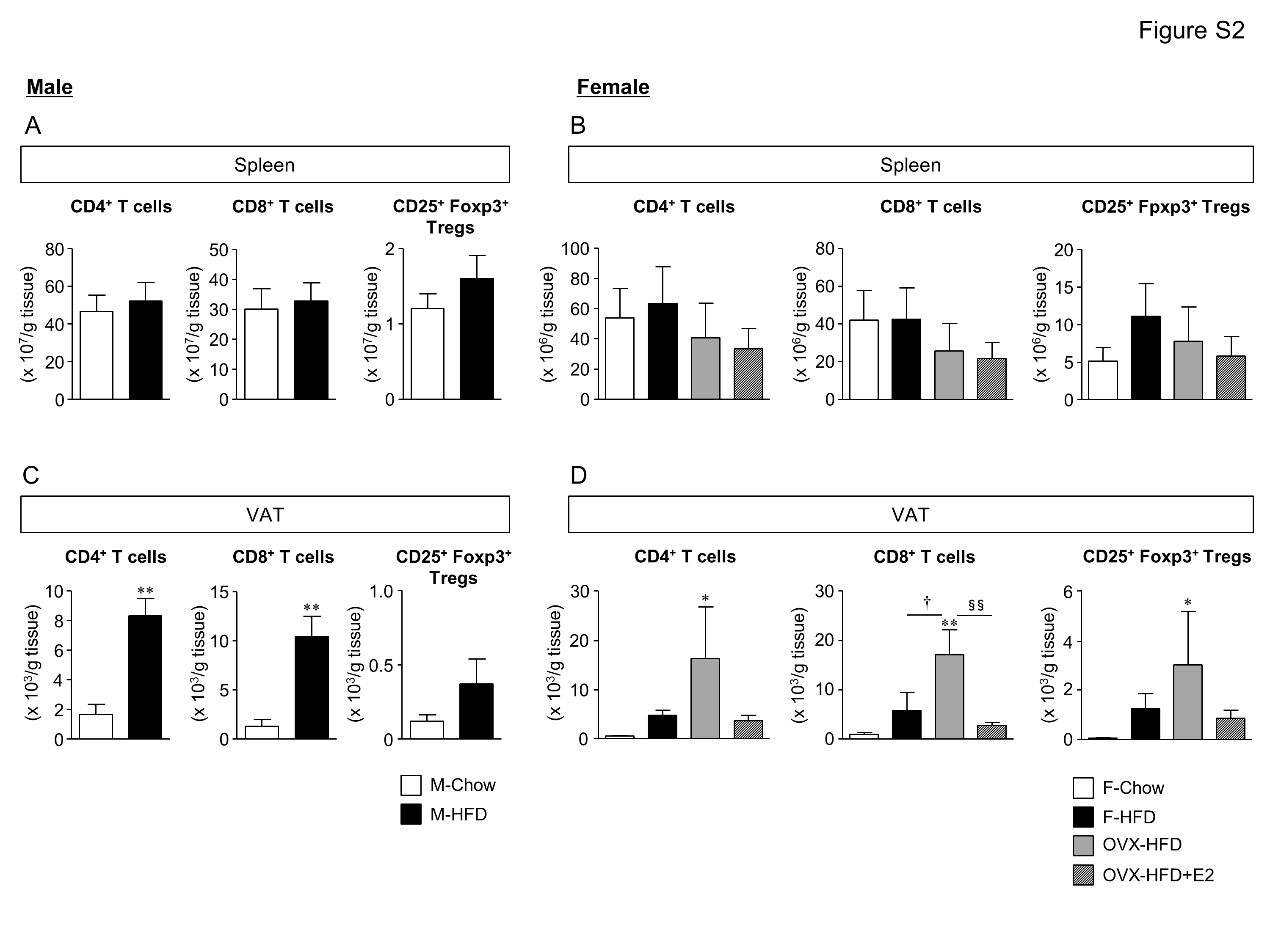

Supplement: S2 Fig — The absolute cell number ratios of CD4+ and CD8+ T cells and CD4+CD25+FOXP3+ Treg in the spleen and gonadal white adipose tissue (Wg) of male and female mice examined by flow cytometry are shown. The results were obtained in the same experiments as in Fig 3. Data are the mean ± S.E. (Spleen n = 5–9, Wg n = 4–6). *P<0.05 and **P<0.01, significantly different from control mice; **P<0.01, significantly different from control mice; †P<0.05, significantly different between F-HFD and OVX-HFD mice; §§P<0.01, significantly different between OVX-HFD and OVX-HFD+E2 mice. (TIF) [file pone.0230885.s002.tif]

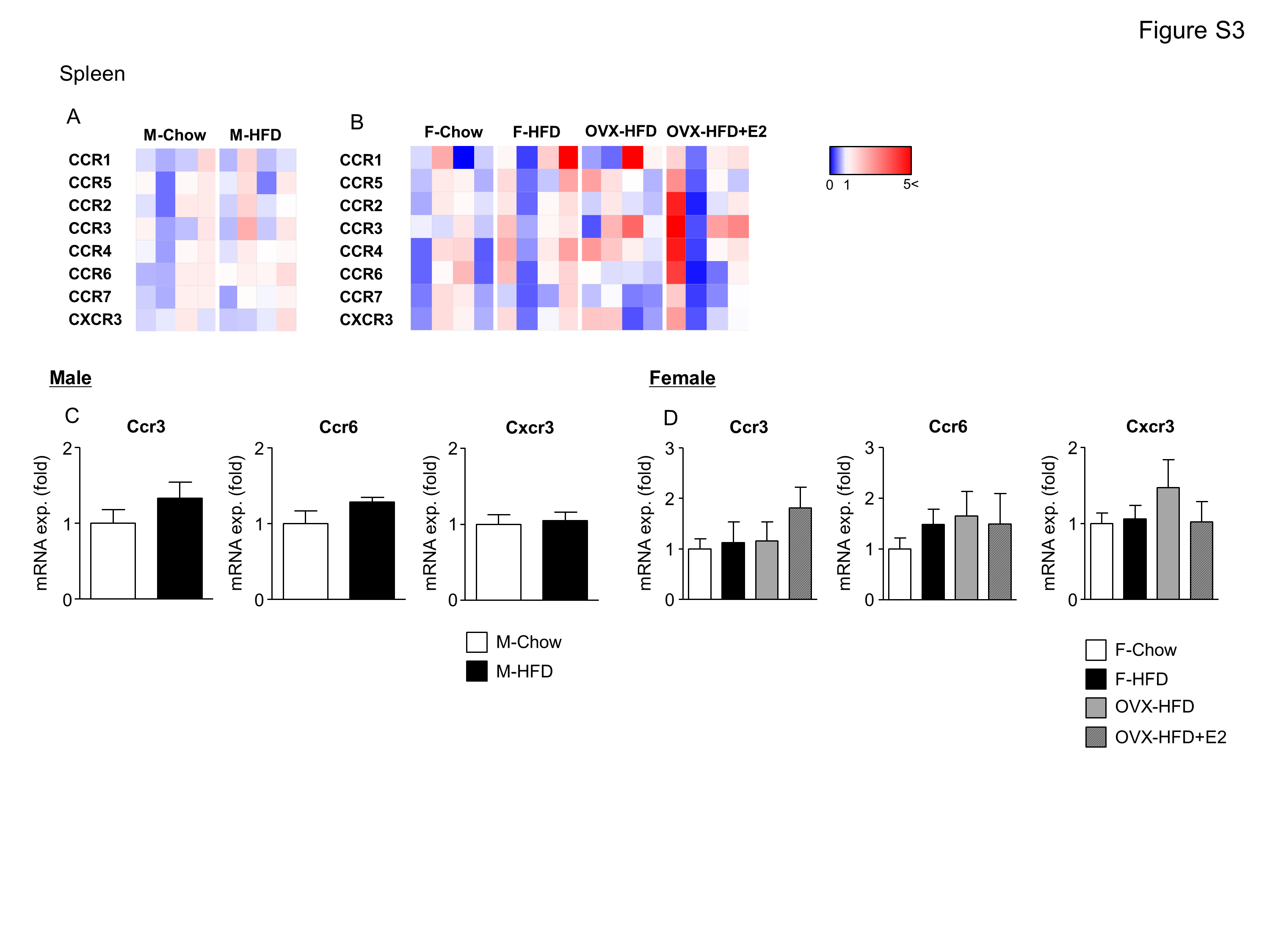

Supplement: S3 Fig — CD4+CD25+ T cells were isolated from spleens of male (A, C) and female (B, D) mice by FACSAria cell sorter. mRNA expression of chemokine receptors in CD4+CD25+ T cells were analyzed by real-time PCR. Heat map analysis showing similar gene expression pattern in males and females of each mouse group. Color from red to blue indicates high to low expression. mRNA expression of Ccr3, Ccr6, and Cxcr3 in CD4+CD25+ T cells is shown. Data are the mean ± S.E. (n = 4–9; C, D). (TIF) [file pone.0230885.s003.tif]
