# Supplementary material for: Estrogen regulates sex-specific localization of regulatory T cells in adipose tissue of obese female mice
Source: PLoS One. 2020 Apr 2;15(4):e0230885. doi: 10.1371/journal.pone.0230885 (PMC7117686; doi:10.1371/journal.pone.0230885)
Supplement: Attachment — Submitted filename: PONE-D-19-30423 Responce to Reviewers.docx [file pone.0230885.s005.docx]

**Answers to Academic Editor**

The Editor indicated there were major issues identified by the Reviewer 1 that required significant attention, additional experiments, and re-write manuscript. We have carefully considered all of the comments of the Reviewers. We added gating strategy of Tregs (Fig. 1S), flow cytometry data of CD4^+^ and CD8^+^ T cells (Fig. 3), absolute cell counts for flow cytometry data (Fig. 2S), and measured *Il1b* expressions in the Wg of each mouse as another inflammatory cytokine (Fig. 4) in the revised manuscript.

The Reviewer 1 has requested several flow cytometry experiments. We understand analysis with flow cytometry provides more informative and straightforward results compared with mRNA analysis of sorted cells by flow cytometry. Ideally, all experiments should be performed by flow cytometry. However, we could not conduct additional flow cytometry experiments. There are several reasons why we could not confirm our results with flow cytometry, and we answered each of them in the Response to Reviewer 1. We would like to explain an overview here. The main reason is limited number of stromal vascular fraction (SVF) cells available in adipose tissues. The number of SVF cells in adipose tissue is low, especially in lean animals. Therefore, more than one mouse per one sample is needed for each analysis (macrophages, expression of ST2 or one chemokine receptor on Tregs). Our FACSArea II system (BD Bioscience) can measure only 6 colors simultaneously to analyze adipose tissue SVF cells with appropriate compensation. We have already used 5 colors (7AAD, CD45, CD4, CD25, and Foxp3) for Treg separation. In addition, we need strict consideration of experimental ethics regarding the number of mice required for the experiments. Furthermore, it would take 5 month to prepare mice for flow cytometry analysis, because mice were fed high-fat diet for 16 weeks after sham or ovariectomy.

Current study has shown the sex difference on VAT-Treg accumulation in obese condition. We screened mRNA expression of several chemokine receptors, and found synchronous changes in *CCR3, CCR6, and CXCR3* expression on Tregs with VAT-Treg localization. Although we did not confirm their expressions by flow cytometry, we believe that the current findings are novel and meaningful for further understanding the localization of VAT-Treg.

We would like to thank the Editor and the Reviewers for highly evaluating our manuscript. Our detailed response to each of the Reviewer’s criticism is described bellows. We really hope that our revised manuscript is now acceptable for publication in PLOS ONE.

**Answers to Reviewer 1**

We thank the Reviewer for evaluating our manuscript and his/her thoughtful suggestions for improving our manuscript.

1. *The results from Fig 1 to Fig 2 assessing the impact of ovarian hormones and E2 on HFD -induced metabolic phenotypes and on glucose tolerance are largely redundant to previously published works in this field.*

The Reviewer suggested that impacts of HFD feeding, results in Figs. 1 and 2 are redundant, since ovariectomy and estradiol (E2) treatment on body weight gain and glucose metabolism are already reported. Although the Reviewer’s indication is taken, we carefully re-consider that these results are still important basic data for properly understanding current study, because body fat accumulation, adipose Treg accumulation and glucose metabolism are closely related to each other. In addition, we did provide a novel method for E2 supplementation in mice, which was modified from a previous reported study on rats [Reference 15, Horm Behav. 2002 doi: 10.1006/hbeh.2002.1835.]. We administered 1.5 µg of E2 every 4 days to imitate the estrus cycle of mice. Most of metabolic parameters including body and fat weights, energy metabolism, glucose levels in glucose and insulin tolerance test were comparable between female sham-operated HFD-fed mice (F-HFD) and E2-supplemented ovariectomized mice fed HFD (OVX-HFD+E2), indicating that the method of E2 supplementation appears to be adequate in the experiments. Although continuous E2 supplementation methods such as sustained releasing tablets or osmotic pump are commonly used for mice experiments, we believe the current new method is considered simple, cost-effective and physiological approach. Therefore, we understand that demonstration of results in Figs. 1 and 2 is important for verifying the data with the current supplementation method.

2. *The results in Fig 3 suggesting sex differences in the recruitment of Foxp3 Treg cells are interesting but very preliminary. The authors should show the gating strategy they used and provide a complete analysis of others immune cells associated with VAT, particularly ILC2. Sex bias have been shown regarding ILC2 numbers in various tissues (Cephus Cell report 2017; Laffont JEM 2017). They should also express the results as absolute cell numbers normalized to tissue weight.*

The Reviewer indicated that sex differences in the recruitment of Foxp3 adipose Treg cells in Fig. 3 are interesting but preliminary. VAT-Treg plays significant role in the attenuation of obesity-associated chronic inflammation, thereby contributing to the maintenance of systemic glucose metabolism in male animals [Reference 7, Nat Med. 2009, doi: 10.1038/nm.2002.; Reference 8, Nat Med. 2009, doi: 10.1038/nm.2001.]. Therefore, we think that the observation is interesting as the Reviewer indicated. In the following experiments, we attempted to investigate the possible underlying mechanisms of VAT-Treg accumulation in female mice by screening their chemokine receptor expressions, since distribution of resident Treg has been shown to be regulated by chemokine signals in various tissues [Reference 27, Blood. 2006, doi: 10.1182/blood-2006-01-0177.]. We really hope that the Reviewer understands our research focus and strategy.

In response to the Reviewer’s request, we added gating strategy of Treg analysis, which was added as the S1 Fig. and described in the method section of the revised manuscript (page 10, line 145).

The Reviewer suggested to provide complete analysis of other immune cells associated with VAT, particularly ILC2, since their sex bias has been shown recently. Therefore, we demonstrated CD4^+^ and CD8^+^ T cell data in the spleen and gonadal white adipose tissue (Wg) in addition to Tregs in Fig. 3 and results section of the revised manuscript (page 15, line 211 to page 16, line 219), although we could not provide complete analysis of immune cells including ILC2. We understand that the Reviewer’s suggestion about ILC2 analysis is quite interesting, because recent studies have indicated that adipose ILC2 plays significant roles in tissue homeostasis and browning. However, preparation of new separate sets of each mouse group is required for the complete analysis of immune cells including ILC2. It will take about more than 5 month for the preparation of mice, because mice were loaded HFD for 16 weeks after ovariectomy. Since we really understand that the adipose ILC2 analysis is another important research topic in the future, we proposed ILC2 experiment as a crucial and needed topic in the discussion section of the revised manuscript (page 25, lines 362 to 369), as follows: Group 2 innate lymphoid cells (ILC2) have recently been highlighted as immune cells controlled by sex hormones. Male are less susceptible to allergic airway inflammation, have a lower prevalence of asthma, and have lower ILC2 numbers in the lung and circulation compared with female mice and asthma patients. The negative impact of androgen and testosterone signaling has been suggested as a mechanism for the reduction in males [32, 33]. Since recent evidences suggest that ILC2 play crucial roles in adipose tissue homeostasis and browning [34], it would be interesting to investigate the sex difference of adipose ILC2 in various conditions such as obese or cold stimulation.

The Reviewer suggested to explain the results as absolute cell numbers normalized to tissue weight in Fig. 3. The presentation of flow cytometry data with cell frequency or absolute cell numbers is sometimes controversial. We considered cell frequency data to be useful because of the low variability and elimination of dead cells. In this context, we presented Fig. 3 as the data with cell frequency. In contrast, data with absolute cell number is also important especially for the analysis with rarely localized cell types. Indeed, frequencies of lung ILC2 cells are rare (about less than 1% in both papers: Cephus Cell report 2017 and Laffont JEM 2017). We understand that presentation of data with both frequency and absolute cell numbers is ideal, as the Reviewer suggested. In this context, the absolute numbers of adipose Treg did not change significantly in female F-HF and OVX-HFD+E2 mice and even in any group of male mice, possibly due to remarkable increase of CD4^+^ T cells by HFD feeding in our experimental condition. Since adipose Treg is usually evaluated by frequency data [Reference 7, Nat Med. 2009, doi: 10.1038/nm.2002.; Reference 8, Nat Med. 2009, doi: 10.1038/nm.2001.] and the interpretation of absolute cell numbers data is difficult, we added these data in the S2 Fig. of the revised manuscript.

3. *The rationale of the experiment in Fig. 4 is unclear. Only TNFa is a pro-inflammatory cytokine gene. Emr1 and Itgax encode for F4/80 and CD11c, respectively. The expression of F4/80 and CD11c + cells must be assessed by FACS rather than RT-qPCR to be really informative.*

Obesity-associated infiltration of proinflammatory macrophage is a well-known feature of chronic inflammation in the visceral adipose tissue that produces inflammatory cytokines including TNFα. The Reviewer suggested that only *Tnfa* is a pro-inflammatory cytokine gene shown in Fig. 4 of the original manuscript. Since we agree with the Reviewer’s indication, we analyzed the expressions of *Il1b* as another important cytokine known to be implicated in the development of insulin resistance in the obese adipose tissue. The expression showed similar change compared to *Tnfa* in the mice group. These new results are added in the Fig. 4 and stated in the result section of the revised manuscript (page 17, lines 231 to 232), as follow: the expression of macrophage markers *Emr1*and *Itgax,*and proinflammatory cytokines *Tnfa* and *Il1b* were significantly increased (Fig. 4A-D).

The Reviewer indicated that *Emr1* and *Itgax* encoding F4/80 and CD11c are representative markers for macrophages and proinflammatory M1-macrophages, respectively. The Reviewer suggested to assess these cell numbers by flow cytometry. We understand that analysis with flow cytometry is ideal for evaluation of macrophage infiltration. However, the number of SVF cells in adipose tissue is low especially in lean animals. More than one mouse per one sample is needed only for the macrophage analysis. In addition, it takes more than 5 months to prepare the new separate set of each mouse group for the assay. We really hope that the Reviewer understands the practical difficulties of analyzing adipose tissue with flow cytometry. In contrast, mRNA expressions of *Emr1* and *Itgax* are usually analyzed as adequate indicators of macrophage infiltration in adipose tissue (e.g. Takei R, PLOS One 2019, doi: 10.1371/journal.pone.0223302.; Li J, Nat Commun 2019,  doi: 10.1038/s41467-019-10348-0.; Kawano Y, Cell Metab 2016, doi: 10.1016/j.cmet.2016.07.009.). Therefore, we measured these mRNA expressions as indicators of obesity-associated chronic inflammations in these animals. Again, we really hope that the Reviewer understands our careful decision on the experiments.

4. *Again, the rationale of Fig 5 is also unclear. Il1rl1 (ST2) IL33 receptor expression could be simply assessed by FACS on Tregs….
   IL33 is an alarmin produced by epithelial cells undergoing cell death or necrosis, not by hematopoietic cells. There is no rationale to test IL33 mRNA expression in Treg cells….*

The significance of IL33/ST2 signaling in the localization of VAT-Treg in male mice has been previously demonstrated [References 20, 21, 26 of the revised manuscript]. Therefore, we investigated whether the expression of *Il1lr1* is associated with VAT-Treg localization in female mice, and found that they were not correlated as described in the discussion section. The Reviewer again suggested that investigation of ST2 (*Il1rl1*) expression in Treg could be assessed by flow cytometry rather than mRNA analysis in sorted samples. We really understand the importance of ideal analysis with flow cytometry for ST2 expression in Treg. Again, we would like to mention that the number of SVF cells in adipose tissue is limited. Preparation of a new separate set of samples from each mouse group is practically very difficult for the assay. On the other hand, we believe that the measurement of mRNA expression is an adequate approach for analyzing limited samples with appropriate biological implications.

We measured IL33 levels in the visceral adipose tissue, since it is an alarmin, as the Reviewer indicated. We labeled “VAT” and “CD4^+^CD25^+^ T cells” above panels to indicate analyzed samples in the revised Fig. 5.

5. *In Fig 6 the data must be expressed as relative expression normalized to house keeping genes rather than fold-change which does not mean anything. Again, the expression of chemokine receptor (CCR3, CCR6, CXCR3) could be easily tested by FACS on Treg cells.*

All of expression data including Fig. 6 are expressed as relative expression normalized to the 18S ribosomal RNA, as described in the material and method section.

Current study has shown the sex difference on VAT-Treg accumulation in obese condition. We screened mRNA expression of several chemokine receptors, and found synchronous changes in *CCR3, CCR6, and CXCR3* expression on Tregs with VAT-Treg localization. Although we did not confirm their expressions by flow cytometry analysis, we believe that the current findings are novel and valuable for further understanding the mechanism of VAT-Treg localization. At the same time, we understand the limitation that current study shows a possible involvement of these chemokine signaling in the clarification of VAT-Treg localization mechanisms in female. The limitation of the current study and requirement of the suggested future research has been described in the discussion section of the manuscript. Furthermore, since the Reviewer’s indication that chemokine receptor expressions should be confirmed by flow cytometry analysis is well taken, we included this point in the discussion section of the revised manuscript (page 25, lines 355 to 361), as shown below: As current limited knowledge regarding the molecular mechanism of estrogen and chemokine signaling for Treg mobilization should be further clarified in the future. We screened several chemokines and their receptors, and provided several candidate of chemokine signaling implicating VAT-Treg localization especially in females. It would be important to verify the expression of these receptors on Tregs by analysis with flow cytometry. Furthermore, the impact of chemokine receptor intervention identified in this study on VAT-Treg localization in female mice is needed to be clarified.

**Answers to Reviewer 2**

We thank the Reviewer for highly evaluating our manuscript and his/her thoughtful suggestions for improvement of our manuscript.

1. The Reviewer suggested to revise references in the method section to adequately repeat the experiments for readers. According to the Reviewer’s important suggestion, we have carefully checked all of references regarding the adequate explanation of the experimental protocol in the method section. As a result, we deleted references 16 and 19 in original manuscript, and adequately corrected and added one paper (reference 17) in the revised manuscript.
2. The Reviewer asked to describe fasting time of GTT and ITT experiments. We agree with the Reviewer’s indication, since fasting time is important for understanding of GTT and ITT data. In response to the Reviewer’s comment, we stated the fasting time in the method section of the revised manuscript (page 8, line 115 to page 9, line 117).
3. The Reviewer advised to describe the relative centrifugal force when reporting centrifugation in the method section. According to the Reviewer’s comment, we changed the description about centrifugation from RPM to RCF in the revised manuscript (Page 9, lines 122 and 128).
